# Embryonic ILC-poiesis across tissues

**DOI:** 10.3389/fimmu.2022.1040624

**Published:** 2022-12-20

**Authors:** Daniela Carolina Hernández-Torres, Christina Stehle

**Affiliations:** ^1^ Innate Immunity, German Rheumatism Research Center (DRFZ), Leibniz Association, Berlin, Germany; ^2^ Medical Department I, Charité – Universitätsmedizin Berlin, Corporate Member of Freie Universität Berlin and Humboldt-Universität zu Berlin, Berlin, Germany

**Keywords:** innate lymphoid cells (ILCs), progenitors cells, embryonic development, *in situ* differentiation, prenatal immunity

## Abstract

The family of innate lymphoid cells (ILCs), consisting of Group 1 ILCs (natural killer cells and ILC1), ILC2, and ILC3, are critical effectors of innate immunity, inflammation, and homeostasis post-natally, but also exert essential functions before birth. Recent studies during critical developmental periods in the embryo have hinted at complex waves of tissue colonization, and highlighted the breadth of multipotent and committed ILC progenitors from both classic fetal hematopoietic organs such as the liver, as well as tissue sites such as the lung, thymus, and intestine. Assessment of the mechanisms driving cell fate and function of the ILC family in the embryo will be vital to the understanding ILC biology throughout fetal life and beyond.

## Introduction

Predominantly known as tissue-resident sentinels, innate lymphoid cells (ILCs) are innate lymphocytes that, in large part, share transcriptional identities and effector function profiles with their adaptive immune system counterparts, T cells. The ILC family consists of three primary lineages: Group 1 ILCs, ILC2, and Group 3 ILCs (as reviewed in Vivier et al., 2018) (1). Group 1 ILCs comprise both T-bet^+^ ILC1 and Eomes^+^ natural killer (NK) cells, which are the counterparts of T_H_1 and T_cytotoxic_ cells, respectively, mount an anti-viral and anti-tumor type 1 response and secrete IFNγ. ILC2 express the lineage-identifying transcription factor GATA3, reflecting T_H_2 cells, and are part of a type 2 allergic response through secretion of IL-4, IL-5, and IL-13. Finally, Group 3 ILCs are identified by the hallmark transcription factor (TF) RORγt, and are classified into two subsets: lymphoid tissue inducer (LTi) cells, which are of particular importance during embryonic development, as discussed later in-depth, and ILC3, which are a counterpart to T_H_17 cells and have an anti-bacterial type 3 response through production of IL-17 and IL-22. Whereas adaptive lymphocytes are found primarily in the circulation and lymph tissues, ILCs are mainly tissue-resident and particularly enriched at mucosal surfaces, such as the lung and intestine. There, they play a significant role in regulating tissue homeostasis in the adult, together with their role as regulators of immunity. However, the developmental history of ILCs begins early on in life, even before adaptive lymphocytes develop.

## Circulating ILC progenitors in the embryo

As all lymphocytes, ILCs originate through the process of lymphopoiesis, whereby lymphoid cells arise from hematopoietic progenitor cells (HPCs) in a series of sequentially restrictive steps as they ultimately commit to a single lineage (reviewed in Doulatov et al., 2012; Golub and Cumano, 2013) (2, 3). The first wave of progenitors in the mouse originates within the yolk-sac (YS) from embryonic gestation day 7 (E7) and from the aorta-gonad-mesonephros (AGM) from E10.5. Here, hemogenic endothelial cells transform into cells with hematopoietic potential which travel through the vascular system to colonize the fetal liver, the primary site of hematopoiesis (4–7). The equivalent in humans occurs at around weeks 3-4 of gestation (8). From HPCs rise the common lymphoid progenitor (CLP), which has lost myeloid potential but has the ability to generate ILC subsets, as well as T cells and B cells (9, 10). In the adult, the transition from HPC to CLP in the mouse is dependent on IL-7 (11, 12), a cytokine that plays a vital role in lymphoid development and maintenance, particularly that of ILC2 and ILC3 as shown in *Il7* ^-/-^ mice (13–18). IL-7R-expressing progenitors can be identified in the YS as early as E9.5 (19). Downstream of the CLP, ID2-expressing as well as PLZF^+/-^ precursors have been identified in fetal liver and newly generated mouse strains have highlighted their potential to generate all ILC subsets (17, 20–25). Of note, we will use the simplified term innate lymphoid cell progenitor (ILCP) to describe ILC-restricted precursor populations including subsets that were originally named EILP, αLP or ChILP in the primary publications. Various investigations have highlighted their complex transcriptional network regulating ILC ontogeny during embryogenesis, including lineage-specific transcripts such as *Tbx21, Eomes, Rorc, and Gata3 (* 26–30). Interestingly, these lineage-identifying transcription factors in the fetal ILCP pool are often co-expressed before commitment to a single lineage (21, 31). Although human fetal ILC-poiesis studies naturally lag behind that of the mouse, the era of single-cell analyses has also allowed for fetal landscape interrogation in humans. Human fetal liver samples ranging from 4 to 17 weeks post-conception (PCW) have been assessed by various groups at single-cell resolution (32–34). In these studies, human fetal liver ILCP have been described as a heterogeneous population expressing *KIT, IL7R*, and *KLRB1* (encoding CD161, a human ILC marker). This follows the description of a circulating ILCP in both adult and fetal tissues in 2017 (35). Liu and colleagues further detected low expression of CD34, marking their potential progenitor properties more directly (32). In humans, PLZF is expressed by fetal and circulating ILCP, as well as differentiated ILCs, making it ineffective as a marker of progenitors (32, 35, 36). Interestingly, these circulating ILCP have a substantial type 3 signature reminiscent of group 3 ILCs, including *RORC* transcripts as well as *KIT, LTB, LTA*, and *CXCR5*, as shown in the online data repositories (32, 33). It remains an open question whether there is a transient upregulation of *RORC* starting from the ILCP during human ILC maturation or whether the *RORC*-expressing ILCP described in these studies rather represent progenitors with LTi potential. Specifically, *LTB, LTA*, and *CXCR5*, which encode functional molecules during lymph node (LN) development, have been described in connection with an LTiP signature (21, 31). Future studies are needed to reveal these cell populations’ precise progenitor-progeny relationship and functionality.

## Tissue ILC progenitors in the embryo: A revised view on ILC-poiesis

**Figure 1:**
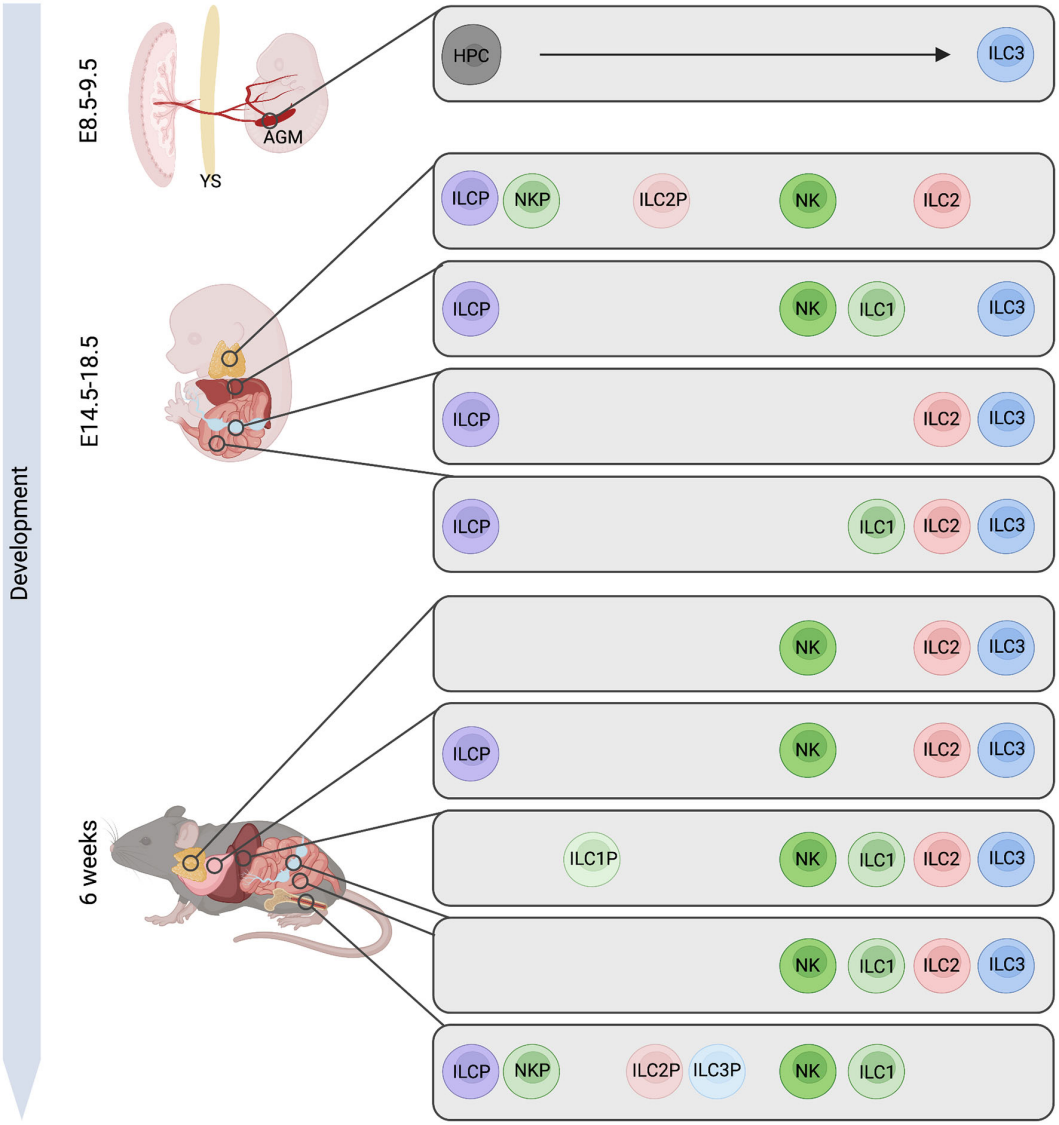
Murine ILC-poiesis from embryo to adult. Early hematopoietic progenitor cells (HPCs) arise from hemogenic endothelial cells within the aorta gonad mesonephros (AGM) region giving rise to ILC3. Later in ontogeny, innate lymphoid cell progenitors (ILCP) are detected in fetal liver but also in lymph node (LN) Anlagen or small intestine, while this population in the adult has only been described in the bone marrow. Further, precursors to specific ILC lineages (NKP, ILC1P, ILC2P, ILC3P) are present in the embryonic thymus, in fetal liver and LN while these cells are mostly confined to the adult bone marrow with exceptions of ILC1P and ILC2P also being found in liver and lung tissue. Figure created with BioRender.com.

The overhanging dogma regarding circulating ILC progenitors was that they home to the bone marrow (BM) from the fetal liver shortly before birth, where they remain into adulthood as the primary site of hematopoiesis. However, multiple studies over the past years have suggested that ILCs arise from fetal ILCP in distinct waves of differentiation throughout embryogenesis, as summarized in **Figure 1**. ILC layered ontogeny generates specialized tissue subsets from local ILC progenitors as shown in the lung, thymus, intestine, or liver (31, 37–42). This suggests that ILC progenitors may seed tissues during embryogenesis and that hematopoiesis in the embryo is not restricted to the fetal liver or BM. In 2015, Bando et al. showed a fetal intestinal precursor expressing Arginase 1 (encoded by *Arg1*) that has the capacity to give rise to ILC1, ILC2, and ILC3, suggesting a model where fetal progenitors may seed peripheral organs and differentiate *in situ* during embryogenesis (43). This fetal progenitor seeds the intestine at day E13.5, prior to Peyer’s patches organogenesis. By single-cell RNAseq analysis, we have recently identified progenitors with CLP-like signatures and an IL-7Rα^+^ α4β7^+^ PLZF^hi^ ILCP in the fetal intestine, likely at least partially overlapping with the Arg1^+^ ILCP described by Bando et al. ( 43). The transcriptional profile of this fetal ILCP correlated with that reported in the BM and fetal liver and included ILCP-associated genes *Zbtb16, Tcf7, Tox, Id2*, and *Arg1*, as well as ILC-lineage transcripts such as *Rora, Ahr, Il7r, Il2rb*, and *CXCR6 (* 31). Similarly, CLP, as well as ILCP populations, could be found in the embryonic periphery, enriched in LN Anlagen (39). In the embryonic thymus, studies have revealed a first wave of early thymic progenitors (ETPs) from E13 and E15.5 capable of giving rise to ILCs as well as T cells (19, 40). In concordance, progenitors with T cell and ILC potential have been described using single-cell *in vitro* cultures of sorted early T cell progenitors from E13 thymi on OP9 stromal cells (19). *In vivo* adoptive transfer of E13 ETPs into irradiated neonatal *Rag2* ^-/-^ *Il2rg* ^-/-^ mice produced all ILC lineages as assessed five weeks later in the intestine of host mice, showing their potential to generate innate lymphocyte lineages. Together, these studies show that tissue-specific progenitor seeding gives rise to differentiated ILCs throughout embryogenesis, where they remain in their tissue niche or have the potential to seed other tissues. As such, tissue progenitors have been described in lung tissue of adult mice by Zeis et al. and Ghaedi et al., that also investigated postnatal day 4 and 12 (44, 45). These tissue progenitors were found to express IL-18R, α4β7 and PD1 and presented gene expression of *Tcf7, Zbtb16, Rora* and *Gata3*, thereby displaying core similarities with BM-derived ILCPs. Importantly, a similar population was also identified in human blood and lung (45).

Interestingly, the significant presence of human fetal lymphoid progenitors (CD34^+^ CD127^+^) in the fetal intestine suggests it is a site of hematopoiesis in the human as well (32). Liu and colleagues analyzed human fetal tissues from 8, 10, and 12 weeks PCW, specifically the liver, thymus, spleen, intestine, skin, and lung, and followed the heterogeneous CD34^+^ progenitor pool at single-cell resolution (32). To which extent these cells might resemble the CD34^+^ ILC- and ILC3-committed progenitors previously characterized in human tonsil and intestine need to be ascertained (46, 47). In contrast, the CD34^-^ ILCP described by Lim et al. in adult tissues lack genes associated with general hematopoiesis such as *CD34*, and instead express genes shared with fetal ILCP such as *GATA3, CD7* and *IL7R*, which are also expressed by differentiated ILCs (32, 35). Comprehensive analyses of the differential potential of these precursors, consisting of functional *in vitro* assays, *in vivo* transfers, and in-depth transcriptomic and chromatin accessibility comparisons are required to untangle the differentiation stages of human fetal ILCs.

## Tissue colonization of embryonic ILCs

### NK/ILC1

The main site of NK cell development in adult mice and humans is the BM. However, in the murine fetal liver, the emergence of CD122^+^ progenitors at E13.5, which upregulate DX5 and NK1.1 at E15.5, has been reported (48, 49). By E15/16.5, these cells are suggested to be functionally competent as assessed by cytotoxicity chromium release assay against YAC-1 target cells, or NK1.1-activation and subsequent degranulation. In OP9 co-cultures of E14.5 progenitors, these cells efficiently generated not only NK cells but also T cells *in vitro*, suggesting that these progenitors remain bipotential (48). In addition, it has recently been demonstrated that the fetal liver harbors a Lin^-^ Sca-1^+^ Mac-1^+^ (LSM) population which largely expresses the CLP-associated markers CD34 and Flt3 and that in the adult liver includes CD122^+^ CD49a^+^ cells (50). Although T-bet or Eomes expression were not assessed, these cells are suggested to give rise to ILC1, a process that depends on an IFNγ-feedback loop from NKp46^+^ cells (50). Along these lines, two recent studies utilizing inducible fate mapping models and *in vivo* transfers have also identified an embryonic wave of ILC1 generation (41, 42). Chen et al. found that ILC1 are present at E16.5 in fetal liver with highly cytotoxic Ly49E^+^ subsets residing into adulthood in contrast to Ly49E^-^ ILC1 that are gradually replenished by hematopoietic progenitors after birth (41), in concordance with earlier reports by Filtjens et al. ( 51). Accordingly, T-bet^+^ Eomes^-^ ILC1 were detected in the E16 fetal liver predominating over NK cells with promiscuous DX5 expression, which is subsequently lost after birth (52). During neonatal infection with murine cytomegalovirus (CMV), embryonic-derived ILC1 were potent IFNγ-producers and Ly49E^+^ subsets displayed higher cytotoxic molecules perforin and granzyme B reducing viral loads in adoptive transfer settings into *Tbx21* ^-/-^ mice (41). Besides the fetal liver, the embryonic thymus has also been identified to harbor Group 1 lineage precursors defined as Id2^+^ Bcl11b^-^ as early as E13.5. These cells expressed Eomes and perforin after *in vitro* culture in IL-2/IL-5/IL-18 (40). Group 1 ILCs displaying transcripts of *Klrb1c, Klrk1, and Tbx21* have been described in parallel through a detailed kinetic of thymic cells from E12.5 to postnatal day one at single-cell resolution, suggesting that ILC1-like cells are particularly present at early time points (E12.5-E15.5) (53). Moreover, as ILC1 also reside in the adult intestine, they can be readily detected at E18.5 in the fetal intestine, possibly originating *in situ* from the intestinal PLZF^+^ ILCP (31). In humans, CD3ε^-^ CD56^+^ cells can be detected as early as 6 PCW, gradually increasing in the human fetal liver and in fetal spleens of 15-24 PCW (54). Between 18-36 PCW, NK cells are frequently represented not only in the liver but also in the lung and spleen, and to lower frequencies in fetal BM and mLN, and display organ-specific variations in killer-cell immunoglobulin-like receptor (KIR) expression (55). NK cell-restricted progenitors defined as CD34^+^ CD38^+^ cells expressing CD45RA, CD10, and CD7 but lacking CD127 were found in human fetal liver from gestational week 6 onwards, and also in fetal BM from gestational week 12 (56). Further extending our knowledge on human tissue hematopoiesis, early progenitors defined as CD34^+^ CD38^dim^ with NK lineage potential have also been detected in the fetal human thymus (54, 57). Interestingly, CD127^+^ ILC1, as analyzed by the lack of ILC2- and ILC3-specific surface markers (CRTH2^-^ CD117^-^), are preferentially found in the embryonic thymus but also in the spleen, skin, lung, intestine, and liver with tissue-preferred distributions of subsets (32). Of note, since Eomes transcripts were ubiquitously expressed in Group 1 ILCs, distinct NK/ILC1 identities could not be clearly dissected. Within the embryonic intestine, frequencies of ILC1 in humans were very low compared to adult gut (58–60). However, due to the unclear differentiation of NK and ILC1 in particular tissues, during inflammation, or in general in humans, further investigations focusing on their identities are necessary to tease out fetal developmental pathways. Regarding type 1 immunity, the placental-fetal interface represents a protective barrier. Still, infection with a variety of pathogens inducing type 1 responses, including viruses such as CMV, bacteria like *Listeria monocytogenes*, or the intracellular parasite *Toxoplasma gondii* may cause vertical transmission (61). In the case of CMV infection, NK cells isolated from cord blood of newborn children with congenital CMV infection showed increased expression of cytotoxic molecules granzyme B, perforin, and granulysin, suggestive of *in utero* activation (62). Notably, NKG2C, LILRB1, and CD57 – hallmarks of CMV infection in adults – were lowly expressed in all infants, including non-infected controls, possibly due to a lack of proinflammatory cytokines IL-12 and IL-15, as reported to be reduced in neonatal DC populations (63). For granzyme C-expressing ILC1, a perforin-dependent lethal immunopathology affecting the salivary gland and liver of neonates has been suggested under enhanced STAT5 signaling (64).

### ILC2

The tissue-resident adult pool of ILC2 has been shown to be composed of cells originating from several phases during ontogeny (37). Id2 and Arg1 reporter mice, in combination with complementary fate-mapping methodologies, revealed that tissue ILC2 are generated in three primary waves. In this study, Thy1^+^ IL-7R^+^ ST2^+^ cells were detected as early as day E17.5 in fetal lung, skin, and small intestine, with the majority being replaced by eight weeks of age with tissue-specific signatures of *de novo* replacements. Early postnatal differentiation waves generated the majority of ILC2 in lung and adipose tissue establishing tissue-specific pools of ILC2. Interestingly, the BM did not display any fetal-derived ILC2 suggesting that this niche is only populated post-natally. However, whether peripheral ILC2 in adults derive from distinct fetal or adult hematopoietic stem cell progenitors remains unknown. Interestingly, tissue signals implicated in ILC2 differentiation and maturation have been described in the fetal and adult mesentery where PDGFRα^+^ gp38^+^ mesenchymal cells support terminal ILC2 differentiation from the ILCP but not CLP stage (65). Through single-cell sequencing of E15.5 and E19.5 CD44^+^ CD25^+/-^ cells, ILC2-precursors were detected in the developing embryonic thymus, where RORα expressed by a common progenitor has been identified as a critical checkpoint in controlling T cell versus ILC2 commitment within the sorted double-negative stage 1 and 2 compartments. ATAC-seq and RORα-ChIP-seq analyses revealed the binding of RORα to ILC2-associated genes such as *Il5*, *Il13*, and *Arg1*, as well as to regulatory elements of the loci regulating Id2 and Nfil3, restricting T cell commitment while promoting ILC2 development. In two experimental settings that used either adoptive transfer or ectopic E15.5 thymi transplantation under the kidney capsule, thymus-derived ILC2 are able to migrate to the intestine of host *Rag2^-/-^Il2rg* ^-/-^ mice exposing their capability to seed extrathymic tissues (40). Together, these data expose a common thymic precursor that has the potential to generate T cells as well as ILC2 that seed the periphery. Accordingly, the human embryonic thymus harbors a substantial fraction of ILC2, while lower frequencies can be found in the skin, lung, liver, and intestine (32). Pooling CRTH2^+^ ILC2 from each organ at various time points further exposed a CCR9^+^ Bcl11b^+^ subset, a T cell gene-enriched population (paralleling the murine studies of Ferreira et al., 2021) (40), as well as precursor cells which, however, lack functional validation. In contrast to adult tissues, ILC2 are readily detected in human fetal mLN and intestine as well as in lung (66, 67). The functions of fetal ILC2 have not been suitably addressed; nevertheless, an interesting study by Saluzzo et al. reports a postnatal influx of ILC2 into lung tissue due to first breath-induced tissue damage and its associated IL-33 epithelial response, resulting in alveolar macrophage polarization into a regulatory M2 phenotype and increased resistance to *Streptococcus pneumaoniae (* 68). This perinatal IL-33/ILC2 axis has also been reported to confer a risk for developing asthma later in life if neonatal allergen exposure in the developing lung, such as house dust mite exposure, leads to hyperactivation enhancing type 2 immunity involving not only ILC2, but also dendritic cells as well as Th2 cells (69). Lastly, early-life respiratory viral infections such as infection with respiratory syncytial virus (RSV) and rhinovirus (RV) may also cause ILC2 activation and long-term lung alterations and lead to immunopathology (reviewed in Fonseca et al., 2021) (70).

### ILC3

ILC3 are amongst the earliest lymphocytes to develop during embryogenesis, and include LTi cells which play a fundamental role in the development of lymphoid tissues prenatally. In addition to RORγt, murine LTi cells express CD4 on their surface, as well as functional molecules such as α4β7, CXCR5, RANKL, or CCR6 enabling them to interact with stromal cells to orchestrate LN development (71–73). CD127^+^ CD4^+^ RORγt^+^ LTi cells are detected as early as E12.5 in murine cervical LN anlagen and can also be found in other peripheral LN anlagen, fetal liver, spleen, and intestine (39, 74–77). Challenging the fetal liver as the origin of ILC3 lineages within the embryo, the group of van de Pavert observed that hematopoietic progenitors arise in the hemogenic endothelial sites by E7.5-9 by using *Cxcr4* and *Cdh5* fate-mapping models (39). Interestingly, they furthermore note that in contrast to peripheral LN sites, progenitors and LTi cells within the fetal liver had no direct connection in ontogeny, suggesting that LTi maturation occurs *in situ*. In line with these findings, we observed the presence of an LTi lineage-skewed progenitor population together with mature progeny in the embryonic intestinal tissue (31). Over the last years, several factors influencing the differentiation program of ILC3 have been described, and their spatial availability might control specific maturational steps at different locations. In the fetal liver, Notch signaling has been proposed to regulate the expression of Id2 within progenitors while subsequent expression of RORγt is inhibited by Notch, blocking final hepatic maturation (75). Along these lines, Golub and colleagues suggest that BM-derived CLP from adult mice depend on Notch signaling in the periphery to mature into RORγt^+^ cells but are dispensable for the development of fetal RORγt^+^ ILCs (78). Additional data exposed that, contrary to the fetal liver, developing progenitors in the periphery respond to maternal-derived retinoids to upregulate RORγt, and retinoic acid signaling even controls the size of the secondary lymphoid organs (79). Of note, not only RORγt expression in ILCs is regulated by retinoic acid signaling, but also the expression of the chemokine CXCL13 by stromal lymphoid tissue organizer cells to attract CXCR5^+^ ILC3. Altogether this data demonstrates the importance of retinoic acid availability during embryonic life for the organogenesis of lymphoid tissues (80). Along the lines of maternally transferred signals, Agüero and Ganal-Vonarburg et al. describe that, although the embryo develops in a sterile environment during pregnancy, maternal microbial-derived metabolites involving Ahr ligands can be transferred to the embryonic offspring thereby shaping at least the neonatal ILC3 pool (81). Notably, while Ahr regulates apoptosis and IL-22 production in ILC3 isolated from adult mice, there is no main effect on fetal intestinal ILC3 (82). Moreover, IL-7 is known to play a key role in LN organogenesis by not only regulating the pool size of IL-7R^+^ LTi cells, but also their Lymphotoxin (LT) α1β2 production (83–87). Besides the aforementioned tissues, comparably to ILC1-like and ILC2 subsets, ILC3 could also be detected by analysis of E16 thymic organ cultures (88). However, whether the cells analyzed originated from mature ILC3 lineages or from immature progenitors within the embryonic thymus has not been addressed in this study. Similar observations to those in mice have also been found in human fetal mLN, liver, and intestine by Teichmann and colleagues, who elegantly describe ILCP and LTi compartments with profound corresponding transcriptional profiles and in addition, using single-molecule fluorescence *in situ* hybridization, place *CXCR5* and *RORC*-expressing LTi cells adjacent to *CXCL13*-expressing lymphoid tissue organizer cells in proximal gut mucosa (89). Spatially, human CRTH2^-^ CD117^+^ ILC3 among Lin^-^ CD34^-^ CD45^+^ cells can be detected in liver, intestine, spleen, skin, lung, and thymus at 12 PCW displaying LTi-related as well as non-LTi gene expression patterns in concordance with flow cytometric analysis by Marquardt et al (32, 90). Of note, NKp44 expression was only observed from week 12 onward in the intestine and liver (90–92). Importantly, as CD4 is not expressed by human LTi cells (93), Nrp-1 has been suggested to identify human but also mouse LTi cells instead (94, 95). As such, Nrp-1-expressing CD117^+^ ILC3 can be detected in human fetal gut, mLN and spleen (94). Tissue seeding of ILCs is initiated already during fetal development, which on the one hand facilitates balanced homeostatic conditions early in ontogeny, and on the other confers the first line of defense before the adaptive immune system is generated. In murine neonates, it has been suggested that ILC3 promote IL-22-mediated resistance to pneumonia induced by intranasal challenge with *Streptococcus pneumoniae*, an effect which is dependent on postnatal colonization by commensal bacteria (96). Similarly, in the neonate, Oherle et al. show that lung ILC progenitors can differentiate towards ILC3 *in situ*, and importantly, that this process is dependent on tissue signals (38). Using lineage-specific IGF1 or IGF1 receptor ablation, they showed that IGF1 producing alveolar fibroblasts generate a special niche in the neonatal lung to influence differentiation of PLZF^+^ ILCP thereby regulating ILC3 differentiation including IL-22-mediated immunity in the lung (38).

Characteristic markers of tissue-derived ILC progenitors are listed in **Table 1**.

**Table 1:** Murine tissue ILC progenitors and identification strategies.

Tissue	Time point	Cell type	Identification strategy	Reference
Liver	E13/14/15/17 6-12 w	ILCP ILC1P	Flt3^-^Id2^+^IL-7R^+^Kit^+^α4β7^+^PD1^+^TCF1^+^PLZF^+/-^ Lin^-^Sca-1^+^Mac-1^+^CD122^+^CD49a^+^	(17, 20, 21, 97) (50)
Intestine	E13.5/18.5	ILCP	Flt3^-^Id2^+^IL-7R^+^α4β7^+^PD1^+^PLZF^+^RORα^+^Arg1^+^	(31, 43)
LN anlage	E13.5/15.5	ILCP	Flt3^-^Id2^+^IL-7R^+^ Kit^+^α4β7^+^PD1^+^PLZF^+^RORα^+^	(31, 39, 97)
Thymus	E13.5 E13.5 E14.5	ILCP NKP ILC2P	CD44^+^CD25^-^Id2^+^ CD44^+^CD25^-^Id2^+^Bcl11b^-^ CD44^+^CD25^-^Id2^+^Bcl11b^+^GATA3^hi^RORα^+^ICOS^+^	(40) (40) (40)
Lung	d 4, 6-12 w	ILCP	IL7R^+^IL-18R^+^α4β7^+^ST2^-^CD103^-^PD1^+^PLZF^+^TCF1^+^	(44, 45)
Bone marrow	6-12 w	ILCP NKP ILC2P ILC3P	Flt3^-^Id2^+^IL-7R^+^ Kit^+^α4β7^+^PD1^+^TCF1^+^PLZF^+/-^ Flt3^-^Id2^+^CD122^+^IL-7R^+^CD244^+^CD27^+^ Flt3^-^Id2^+^IL-7R^+^α4β7^+^Sca1^+^CD25^+^ST2^+^Bcl11b^+^ Flt3^-^Id2^+^IL-7R^+^α4β7^+^CD25^-^	(17, 20, 21, 23, 25, 74, 98–100) (101, 102) (103) (23, 75)

Of note, the original publications might use a different nomenclature while we used the standardized term ILCP.

## Unresolved questions and future directions

Several aspects of ILC-poiesis in the fetus remain to be clarified. Revealing the embryonic developmental timing of various ILC populations (**Figure 1**) in particular will require advanced lineage tracing techniques and sensitive mouse models. The exact waves of ILC ontogeny in various organs for example, as well as their contribution to shaping organ architecture and innate immunity post-birth, remains unclear. Whether diverse embryonic waves exclusively produce heterogeneous ILC subsets that remain in the adult potentially locating to specific sites, as shown for innate-like lymphocytes such as B-1a B cells in the peritoneal and pleural cavities, or certain γδ T cell subsets in gut and skin, remains to be addressed in future studies. In this context, it will be an important area of further investigation to reveal the impact of embryonic lymphocyte waves not only on the establishment of immune function and disease susceptibility but also its impact on the development of the physiological functioning of various organs. As *in vitro* cell differentiation and cultivation technologies are rapidly evolving, so does the ability to address the main questions of ILC ontogeny also in humans. The use of comprehensive platforms for *in vitro* ILC generation from CD34^+^ progenitors from various tissues (104), which allow for an accessible resource of ILCs for interrogation and genome editing, as well as the complex microphysiological systems becoming available (105), would allow for the dynamic investigation of human lymphoid developmental pathways and their regulators within tissue niches. As disruption of ILCs and their development can have enduring consequences on the immunity and health of the host, it is critical to understand local tissue ILC progenitor differentiation and maintenance, and the tissue signals which regulate these processes.

## Author contributions

All authors listed have made a substantial, direct, and intellectual contribution to the work, and approved it for publication.
